# Airway surface liquid pH is not acidic in children with cystic fibrosis

**DOI:** 10.1038/s41467-017-00532-5

**Published:** 2017-11-10

**Authors:** André Schultz, Ramaa Puvvadi, Sergey M. Borisov, Nicole C. Shaw, Ingo Klimant, Luke J. Berry, Samuel T. Montgomery, Thien Nguyen, Silvia M. Kreda, Anthony Kicic, Peter B. Noble, Brian Button, Stephen M. Stick

**Affiliations:** 1 School of Paediatrics and Child Health, University of Western Australia, Subiaco, Perth, WA 6008 Australia; 2 Department of Respiratory Medicine, Princess Margaret Hospital for Children, Subiaco, Perth, WA 6008 Australia; 3 Telethon Kids Institute, University of Western Australia, Subiaco, Perth, WA 6008 Australia; 4 Institute of Analytical Chemistry and Food Chemistry, Graz University of Technology, Graz, 8010 Austria; 5 School of Medicine, University of North Carolina, Chapel Hill, NC 27599 USA; 6 School of Anatomy, Physiology and Human Biology, University of Western Australia, Crawley, Perth, WA 6009 Australia; 7 Centre for Neonatal Research and Education, School of Paediatrics and Child Health, University of Western Australia, Crawley, WA 6009 Australia

## Abstract

Modulation of airway surface liquid (ASL) pH has been proposed as a therapy for cystic fibrosis (CF). However, evidence that ASL pH is reduced in CF is limited and conflicting. The technical challenges associated with measuring ASL pH in vivo have precluded accurate measurements in humans. In order to address this deficiency, ASL pH was measured in vivo in children using a novel luminescent technology integrated with fibre-optic probes. Here we show that ASL pH in children with CF is similar to that of children without CF. Findings were supported by highly controlled direct pH measurements in primary human airway epithelial cell culture models, which also suggest that the potential ASL pH gradient produced by defective apical ion transport is balanced out by paracellular shunting of acid/base. Thus, reduced baseline ASL pH is unlikely to be an important pathobiological factor in early CF lung disease.

## Abstract (web summary)

Modulation of airway surface liquid pH has been proposed as a therapy for cystic fibrosis, but whether pH is indeed altered in cystic fibrosis is controversial. Here, the authors develop a novel fibre-optic based pH measurement technology, and show that pH is not altered in children with cystic fibrosis.

## Introduction

Cystic fibrosis (CF) is one of the most common lethal genetic diseases, affecting ∼30,000 people in the United States and over 70,000 people worldwide^1–4^. Mortality is predominantly caused by progressive airway disease resulting in respiratory failure^5^. CF airway disease results from dysfunction of the CF transmembrane regulator (CFTR) protein located on the apical surface of airway epithelial cells where it functions as an anion channel, regulator of other epithelial transport proteins, e.g., the epithelial sodium channel, and mediator of intracellular signalling pathways^6, 7^. CFTR-mediated transepithelial chloride and sodium transport are important for the regulation of airway surface liquid (ASL) hydration and mucociliary clearance^6^ and there is evidence that impaired CFTR-related bicarbonate transport results in reduced ASL pH in CF^8–13^. Tight regulation of ASL pH has been implicated in epithelial cell and ciliary function, mucin formation, optimal function of antimicrobial peptides and leucocyte-mediated bacterial killing (although the influence of ASL pH has not yet been tested on *Pseudomonas aeruginosa—*an important target for antimicrobial therapy^13–16^). Acidic ASL pH may accommodate infection by increasing pro-inflammatory properties of bacterial cell wall components and decreasing bactericidal activity of antibiotics^17–19^.

A controversial issue is whether CF lung disease is initiated by a CFTR-mediated reduction in ASL pH as a result of reduced bicarbonate transport. The most compelling evidence for reduced ASL pH in CF is from newborn CF pigs (ASL pH (mean±s.e.m.) 6.94 ± 0.05 compared to 7.14 ± 0.04 in non-CF littermates)^12^. The lower pH in CF was associated with impaired bacterial killing that was restored by raising ASL pH^12^. Since bacterial infection is a significant driver of CF lung disease progression, this study strongly supported the hypothesis that reduced ASL pH is central to disease pathogenesis in CF.

The in vivo ASL pH measurements in humans have been inconclusive. Nasal pH measurements suggested reduced pH in neonates with CF compared to non-CF controls; however, this difference was not observed in older children and adults^20^. The only study that measured lower airway pH in children found no difference in ASL pH between children with and without CF^21^. Data from both these studies should, however, be interpreted somewhat cautiously due to factors that limit the accuracy of the pH sensors as detailed in the discussion.

We hypothesised that consistent with previous studies in the neonate pig model of CF, ASL pH in the lower airways is reduced in young children with CF compared to children without CF. We used novel methodology and highly controlled conditions to measure ASL pH in young children. Here, we show that ASL pH in children with CF is similar to that of children without CF. Findings were supported by highly controlled direct pH measurements in primary human airway epithelial cell culture models. In cell cultures that overexpress the H+/K+transporter, ATP12A, which is central to CF-related acidification of ASL^22^, there is no reduction in ASL pH. Only when HCO_3_^−^ is removed from the basolateral medium and replaced with HCO_3_^−^-free HEPES (pH = 7.2) does ATP12A-over-expressing cultures exhibit a reduction of pH in thin-film ASL. Thus, ATP12A-overexpressing cells with physiological concentrations of HCO_3_^−^ in the basal medium exhibit ‘normal’ ASL pH, suggesting that the potential ASL pH gradient produced by defective apical ion transport is balanced out by paracellular shunting of acid/base. Thus, reduced baseline ASL pH is unlikely to be an important pathobiological factor in early CF lung disease.

## Results

### Novel pH-sensitive luminescent dye-based fibre-optic probes

To improve the accuracy of pH measurements of ASL in subjects with CF, we developed novel pH-sensitive luminescent dye-based fibre-optic probes. These optical sensors have a high signal-to-noise ratio which allowed pH to be measured with greater precision than has previously been possible^23–26^ and are highly responsive to pH changes in a biological environment. The design of the probes, with the pH-sensitive luminescent dye embedded in a hydrogel matrix, limited interactions between the dye and ASL proteins which could potentially alter the measured pH values.

### ASL pH measurements in vivo

Measurements of pH were obtained from the right middle lobe bronchi of 67 children, 37 with CF and 30 without CF. Children without CF were undergoing investigation for recurrent or chronic respiratory symptoms. Reliable pH readings were obtained in 51 of the 67 children (30 CF; 21 non-CF): 24 females and 27 males. The median (range) age was 50 (12–78) months in the CF group and 36 (16–86) months in the control group. The study had power of 87% to detect a difference between groups of 0.1 pH units at an α-level of 0.05 in a one-sided *t*-test.

**Fig. 1:**
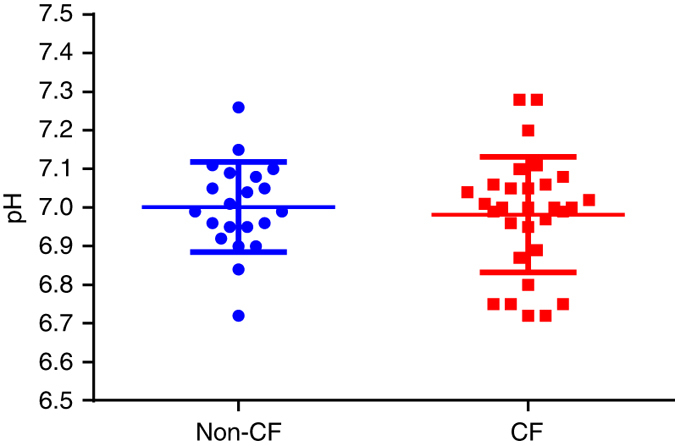
No difference in airway surface liquid pH between children with and without CF. A single pH value was obtained for each individual patient where the mean ± s.d. for non-CF (*n* = 21) and CF (*n* = 30) groups was 7.00 ± 0.12 and 6.98 ± 0.15, respectively. Two-sided *t*-test demonstrated no significant difference between groups (*p* = 0.62). Individual measurements and averaged data are presented as mean ± s.d.

There was no significant difference in ASL pH between groups (Fig. 1) as determined by a two-sided *t*-test, with mean±s.d. pH in CF vs. controls 6.98 ± 0.15 and 7.00 ± 0.12 respectively (*p* = 0.62).

### Lower ASL pH and inflammation

**Fig. 2:**
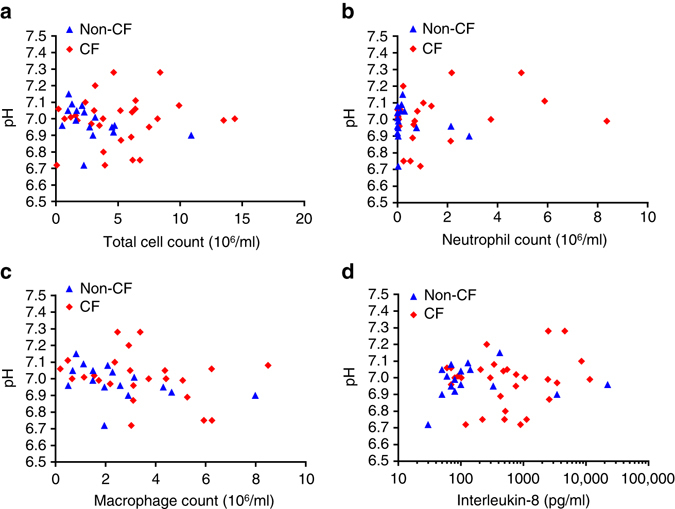
Airway surface liquid pH and inflammatory markers in bronchoalveolar lavage fluid (BALF). **a** No relationship between airway surface liquid pH and total cell count in children with CF (*n* = 29; *p* = 0.83). However, a significant inverse relationship between airway surface liquid pH and total cell count was observed in the non-CF group (*n* = 16; *p* = 0.01). **b** No relationship between airway surface liquid pH and neutrophil count for children with (*n* = 24; *p* = 0.53) or without CF (*n* = 16; *p* = 0.83). **c** A significant relationship between airway surface liquid pH and macrophage count was demonstrated in the non-CF group (*n* = 16; *p* = 0.04) but was not observed in the CF group (*n* = 24; *p* = 0.18). **d** There was no correlation between interleukin-8 (IL-8) levels and airway surface liquid pH for the CF (*n* = 29; *p* = 0.90) or the non-CF group (*n* = 16; *p* = 0.43). For all panels, BALF analysed for inflammatory markers was sampled from the lung lobe corresponding to the airway surface liquid pH measurement. A single pH value was obtained for each individual patient while a single aliquot of BALF was used to measure inflammatory markers. Spearman’s rank correlation test was used for all statistical analyses

In children with CF there was no association between ASL pH and inflammatory markers in bronchoalveolar lavage fluid (BALF) from the same lobe as pH measurements (Fig. 2) by Spearman’s rank correlation test: total cell count (*r* = 0.04, *p* = 0.83), neutrophil count (*r* = 0.13, *p* = 0.53), macrophage count (*r* = −0.28, *p* = 0.18) and interleukin-8 (IL-8) (*r* = −0.02, *p* = 0.90). In children without CF there were significant inverse correlations between ASL pH and total cell count (*r* = −0.61, *p* = 0.01) and macrophage count (*r* = −0.56, *p* = 0.03) but not neutrophil count (*r* = 0.06, *p* = 0.83) or IL-8 levels (*r* = 0.21, *p* = 0.43). Only total cell count (*p* = 0.02) and IL-8 (*p* < 0.01) levels were higher in BALF from children with CF compared to non-CF controls using the Mann–Witney *U*-test.

### Lower ASL pH and infections

**Fig. 3:**
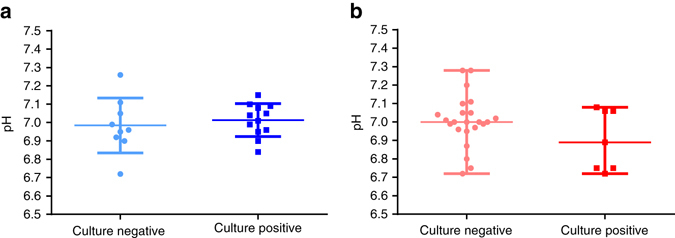
Airway surface liquid pH and infection. **a** Using a two-sided *t*-test to determine statistical significance, there was no difference in airway surface liquid pH between patients who had a positive microbiological bronchoalveolar lavage fluid (BALF) culture result (*n* = 12) compared to patients with a negative culture result (*n* = 9) in the non-CF group (*p* = 0.59). Mean ± s.d. of airway surface liquid pH was 7.01 ± 0.09 and 6.98 ± 0.15, respectively. Data presented as individual measurements and mean ± s.d. **b** A two-sided Mann–Whitney *U*-test demonstrated that there was no difference in airway surface liquid pH between patients who had a positive culture result (*n* = 7) compared to those with a negative culture result (*n* = 23) in the CF group. Median (range) of airway surface liquid pH was 6.89 (6.72–7.08) and 7.00 (6.72–7.28), respectively. Data presented as individual measurements and median (range). For all data points, BALF was sampled from the lung lobe corresponding to the airway surface liquid pH measurement. A single pH value was obtained for each individual patient

There was no significant difference in pH between children who had positive bacterial cultures in BALF obtained from the corresponding lung lobe vs. those who had negative cultures (Fig. 3). Mean±s.d. pH in culture-negative vs. culture-positive groups in children without CF were 6.98 ± 0.15 and 7.01 ± 0.09 respectively (*p* = 0.59) as determined by a two-sided *t*-test. Median (range) pH in culture-negative vs. culture-positive groups in children with CF was 7.00 (6.72–7.28) and 6.89 (6.72–7.08) respectively (*p* = 0.36) using the Mann–Whitney *U*-test.

### ASL pH measurements in vitro

**Fig. 4:**
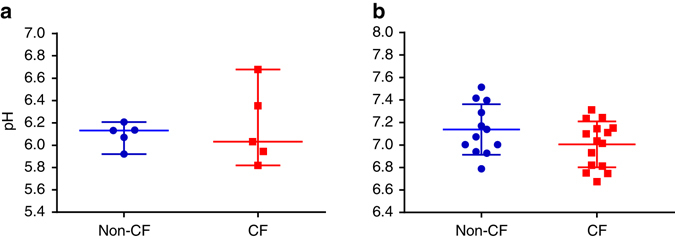
Airway surface liquid pH in airway epithelial cells cultures. **a** No difference (*p* > 0.99) in ASL pH between CF (*n* = 5) and non-CF (*n* = 5) epithelial cell cultures measured using fibre-optic probes. The Mann–Whitney *U*-test was used for statistical analysis. Bars represent median (range). Each of the points represents a different patient (genotypes of CF cultures described in Supplementary Fig. 8). **b** No difference (*p* = 0.12) in ASL pH between CF (*n* = 15) and non-CF (*n* = 12) primary bronchial airway epithelial cell cultures using a conventional (potentiometric sensor) micro-pH probe was found using an unpaired *t*-test. Each of the points represents a different patient (cells harvested from excised CF lung transplant recipient lungs). Bars represent mean ± s.d.

In order to further understand our in vivo observations, ASL pH was studied in primary airway cell cultures using our fibre-optic probes and there was no difference between ASL pH between CF and non-CF cultures (Fig. 4a). Similarly, there was no significant difference between CF and non-CF ASL pH using standard physical, i.e., non-optical, micro-pH probes (Fig. 4b).

**Fig. 5:**
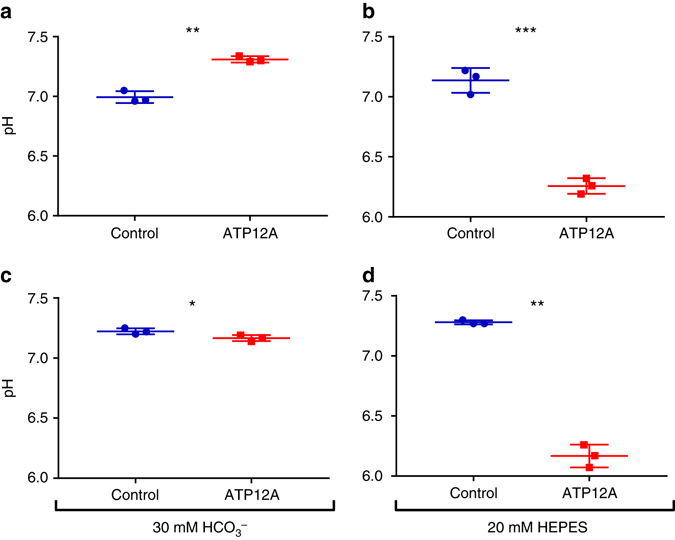
Airway surface liquid pH in ATP12A-overexpressing cells. ASL pH measurements were performed using a microsensor pH electrode in thin-film, unperturbed ASL. Two independent cell models (**a**, **b**) primary human airway epithelial cells and (**c**, **d**) Calu-3 cells were transfected to overexpress ATP12A or empty vector (control cells). **a** In primary human airway epithelial cells with serosal 30 mM HCO3^−^, ASL pH was increased by ~0.31 pH units in ATP12A-overexpressing cells compared to vector controls (*n* = 3; *p* < 0.01). Hence, in the presence of serosal HCO3^−^, overexpressing of ATP12A did not result in ASL acidification. **b** In primary human airway epithelial cells with serosal 20 mM HEPES, ASL pH of ATP12A-overexpressing cells was markedly reduced (~0.88 pH units) compared to vector controls (*n* = 3; *p* < 0.01). **c** In Calu-3 cells with serosal 30 mM HCO3^−^, ASL pH was slightly reduced (~0.06 pH units) in ATP12A-overexpressing cells compared to vector controls (*n* = 3; *p* = 0.04). **d** In Calu-3 cells with serosal 20 mM HEPES, ASL pH was markedly reduced (~1.11 pH units) in ATP12A-overexpressing Calu-3 cells compared to vector control cells (*n* = 3; *p* < 0.01). All statistical analyses were performed using unpaired *t*-tests. Data represented as mean ± s.d.; *p < 0.05; **p < 0.01; ***p < 0.001

A recent report in animal models of CF suggests that the H+/K+transporter, ATP12A, is central to CF-related acidification of ASL^22^. To further explore the effect of ATP12A on ASL, pH was measured in primary human airway epithelial cell cultures that overexpressed ATP12A. The ASL pH of ATP12A-overexpressing cultures was not markedly reduced compared to vector-expressing control cells. Only when basolateral HCO_3_^−^ was removed and replaced with HCO_3_^−^-free HEPES (pH = 7.2) did the ATP12A-overexpressing cultures exhibit a reduction of ~ 1 pH unit in thin-film ASL compared to their respective controls (*p* < 0.01) using a two-sided *t*-test. Thus, ATP12A-overexpressing cells with [HCO_3_^−^] serosal=25–30 mM exhibited ‘normal’ ASL pH which was similar to their control (vector) cells, suggesting that the paracellular path shunts out bicarbonate gradients effectively. These data were supported by independent experiments in an alternative cell model, the airway epithelial Calu-3 cell line^27^ (Fig. 5).

## Discussion

Our novel technology allowed accurate lower airway ASL pH measurements in young children. There were no differences in pH between children with or without CF. These observations were supported by results from experiments with cell cultures using our novel optrodes and classical potentiometric microsensor pH probes. Further experiments with ATP12A-overexpressing cell cultures suggest that the absence of a pH difference between CF and non-CF, in spite of abnormal CFTR-related surface bicarbonate transport, may be explained by paracellular HCO_3_^−^ shunts.

We provide robust evidence that reduced ASL pH is not likely to be a critical factor in the early development and progression of human CF lung disease. The strengths of our study are the novel methodology used that allowed accurate measurements in vivo, inclusion of young CF children that reduced the likelihood of alteration in pH by advanced airway disease (as advanced CF airway disease pathophysiology may not reflect early CF) and measurements performed in children without CF who were relatively healthy, i.e., undergoing routine bronchoscopy in the absence of overt symptoms.

Our results contrast with observations from studies in the porcine model of CF^8, 12^. The contradictory findings in young children vs. newborn pigs could be due to intrinsic physiological factors and our study highlights the need to examine whether there are important species differences when conflicting data arise^28^. Our study was powered sufficiently to detect the differences in pH observed in the studies of pigs. We sampled a relatively large population (30 CF/21 non-CF) that represented the genetic diversity of human CF, whereas the measurements in pigs were from a relatively small number of genetically related CF (*n* = 6) and non-CF (*n* = 8) animals.

A limitation of our study is that we cannot rule out the possibility that glandular secretion caused by mechanical effects on the airway wall influenced our in vivo measurements^13^. However, the 250 μm diameter pH probe used was gently placed under direct vision to minimise ASL disruption and epithelial stimulation. The aforementioned measurements in pigs were obtained using a 3 mm × 3 mm-sized planar optical probe^12^, and therefore mechanical effects of our probes, if relevant, are likely to be far less than those present during measurements undertaken in pigs^29^. Potential mechanical effects in our cell culture measurements would have been less likely, as epithelial cell cultures do not contain glands and the pH probe was advanced in 5 μm increments, with measurements taken as soon as the probe entered the mucus layer which is considerably thicker than 5 μm.

In children with CF, the ASL pH was unaffected by the presence of lower airway inflammation. In contrast, in children without CF, we found weak negative correlations between ASL pH and some markers of inflammation, specifically macrophage numbers. We therefore cannot rule out a small effect of airway inflammation on pH in our control population. However, since inflammation was many fold greater in the children with CF than controls, one would expect this to exaggerate any difference between the groups and further supports the argument that ASL pH is not low in CF.

There were also no differences in pH between children with positive and negative BAL cultures. Others have shown that pH may be altered during CF pulmonary exacerbations and during severe lung infections in people without CF: Tate et al.^30^ observed significant differences in exhaled breath condensate pH between stable CF patients and those with acute pulmonary exacerbations. In addition, non-CF patients with pneumonia were found to have reduced pH in airways from the affected lobe compared to unaffected lobe^19^. Our findings relate to stable CF in children with early disease and we did not observe any associations with pH of inflammation or infection.

The lack of consensus between previous studies examining ASL pH in young children may be explained by technological limitations and/or inadequate sample sizes. McShane et al.^21^ found no differences in lower airway or nasal pH between a group of 5 children with CF and a group of 6 children without CF. Abou Alaiwa et al.^20^ found no difference in nasal pH between children and adults with and without CF, but observed a significantly reduced nasal pH in neonates with CF compared with controls, with a difference in median pH between CF and non-CF of 1.9 units. In both studies, pH probes, with rounded tips and diameters of 2–2.5 mm, were used to perform measurements in the ASL layer that is only a few μm in height. A logical criticism of the oesophageal pH probes is that they would not have been in full contact with the ASL layer, resulting in an integrated signal from the whole surface of the sensor which would have been partly in contact with ASL and partly in contact with air. This technical limitation might, in part, explain the large and non-physiological pH ranges observed in previous studies: e.g., McShane et al.^21^ 5.5 to 7.7 units, and Alaiwa et al.^20^ 4.5 to 7.9 units. Given the large ranges of pH observed and the relatively small sample sizes, there was limited power to detect small differences in pH in these studies.

Inconsistencies in pH measurements between the in vitro cell culture measurements described in this paper (reference Fig. 4a vs. Fig. 4b) and others^8, 9, 12, 13^ are likely to be due to differences in experimental methods. Unlike previous in vitro studies, we performed experiments in a constant carbon dioxide environment to minimise the effects of ambient carbon dioxide variability. We set out to measure ASL pH under steady-state conditions that best represented the in vivo airway microenvironment. Other influential studies have examined dynamic responses to changes in the microenvironment that are non-physiological^8, 9, 13^ or steady-state responses after non-physiological measurement conditions have been imposed^9, 13^. For example, Garland et al.^13^ added 20 µL of phosphate-buffered saline (PBS) to apical cell culture surfaces before measuring pH using dissolved pH indicators without controlling for ambient carbon dioxide levels during optical measurements. Coakley et al.^9^ similarly measured ASL pH after adding a relatively large amount of fluid to the apical cell surface. Numerous factors could potentially have influenced these results: the effect of adding buffer to the apical culture surface is unknown. Changes in ambient carbon dioxide may have influenced results as demonstrated in Supplementary Fig. 1. Such dynamic experiments may not reflect steady state. In contrast, we performed direct measurements in unperturbed, stable systems, with calibrated probes in thin ASL films under highly controlled conditions.

Another factor that would have influenced ASL pH in previous cell culture models of CF is the use of a basolateral buffer solution that did not contain bicarbonate. In ATP12A-overexpressing cell cultures we demonstrated that the paracellular path effectively shunts out bicarbonate gradients. Hence, a lack of bicarbonate in the culture medium could explain why reduced ASL has been found in some previous cell culture studies^8, 12^. The finding that the paracellular path shunts out bicarbonate gradients could also explain why there is no difference between CF and non-CF ASL pH in young children.

In studies where optical pH indicators (dyes) dissolved in the ASL were used for pH measurements^8, 12^, accurate calibration of the pH indicators would have been difficult. Furthermore, interaction of the dye with proteins in the ASL cannot be avoided and is expected to change the calibration of the indicators significantly. Such interactions would be very limited with the optodes used in our study due to the permeation-selective properties of the hydrogel matrix in which the luminescent dyes were embedded.

In conclusion, ASL pH is not reduced in children with CF. The potential bicarbonate and pH gradient caused by abnormal CFTR bicarbonate transport appears to be balanced during steady state by paracellular ion shunting. Our observations therefore do not support the hypothesis that early disease progression is driven by low ASL pH.

## Methods

### Study design

A prospective study conducted at Princess Margaret Hospital for Children. The study subjects were children with CF between 1 and 6 years of age undergoing annual routine bronchoscopy as part of the Australian Early Surveillance Team for Cystic Fibrosis (AREST CF) programme—a clinical pulmonary surveillance programme in which children up to the age of 7 years undergo annual bronchoscopy and BAL for detection of infection. The diagnosis of CF was confirmed by a combination of sweat chloride levels >60 mmol/l, gene mutation analysis and clinical picture, i.e., pancreatic insufficiency. The controls were age-matched children without CF undergoing bronchoscopy for further investigation of recurrent or chronic respiratory symptoms. There was no significant relationship between ASL pH and the age of the patient at the time of measurement in children with (*p* = 0.58) and without (*p* = 0.31) CF as determined by linear regression analysis (Supplementary Fig. 2).

Ethical approval was obtained from the Princess Margaret Hospital for Children Human Research Ethics Committee, and parents of all children recruited gave informed consent.

### Optical pH probe and electronic equipment

**Fig. 6:**
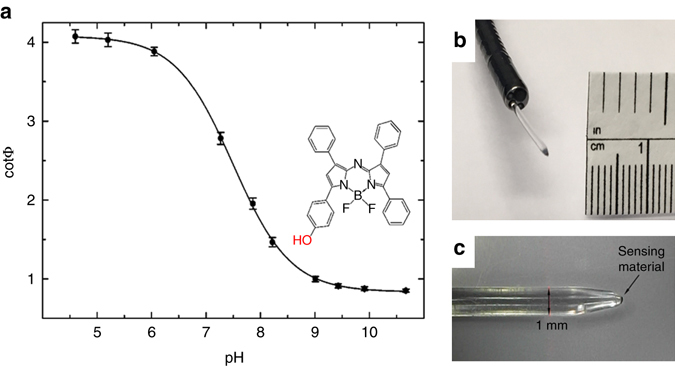
Fibre-optic pH probes. **a** Calibration curves obtained from measurements of three individual sensors at 37 °C and chemical structure of the pH indicator (BF_2_-chelated hydroxy-tetraarylazadipyrromethane^28^). **b** Photograph of the tip of the pH probe protruding from the working channel of a bronchoscope. **c** Photograph of the plastic optical fibre

The pH sensing material relied on the use of BF_2_-chelated hydroxy-tetraarylazadipyrromethane^31^ (Fig. 6) where fluorescence changes as a function of pH (highly fluorescent at low pH and virtually non-fluorescent at high pH). The pH indicator was dissolved in a polyurethane hydrogel D4 (0.4% wt. in respect to the hydrogel). Microparticles of inorganic phosphor Egyptian blue^32^ (pH insensitive) was added to the material (1.3:1 wt. of Egyptian blue/D4) to enable dual lifetime referencing^33, 34^ readout. The composition was coated onto a tip of jacket-free PMMA (poly(methyl methacrylates) fibre of 120 cm in length (Ratioplast, Germany). Prior to coating, the 1 mm fibre was pulled over a hot filament to reduce the tip diameter to ∼250 µm (Fig. 6). A small cavity in the middle of the tip was produced with a hot needle to enhance the adsorption of the sensing material.

The fibre-optic probes were connected to a phase fluorometer (Firesting, PyroScience, Aachen, Germany) which delivered a red excitation light with a wavelength of 624 nm to the sensor and a near-infrared range emission light (700–1,000 nm) back to the photodetector. The luminescence phase shift *Φ* and cotΦ reflected the response of the sensor (Fig. 6a), the latter being proportional to the amount of the pH indicator in fluorescence form^30^. As can be seen (plot of cotΦ vs. pH), the material showed optimal resolution in the range from 6.5 to 8.5 (pKa 7.5).

The sensors possessed slightly varying ratio of the indicator to reference phosphor particles and required calibration prior to use; this was performed using buffers with pH 4.5, 7.6 and 10.5 (acetate, phosphate and 3-(cyclohexylamino)-1-propanesulfonic acid) adjusted to physiologic ionic strength with 150 mM of sodium chloride and immersed in a water bath at 37 °C. Validation of the pH probes for in vivo use is discussed in the Supplementary Information online.

### Lower airway pH measurements

**Fig. 7:**
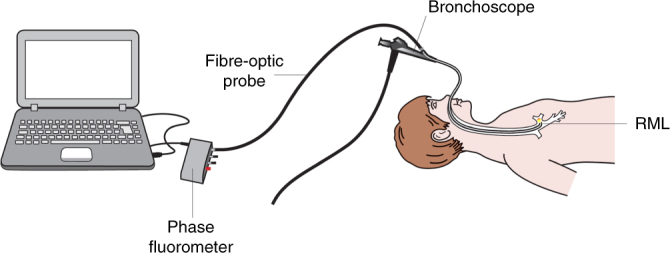
Schematic representation of in vivo transbronchoscopic airway surface pH measurements. RML right middle lobe bronchus

Lower ASL pH measurements were performed from the right middle lobe transbronchoscopically by inserting a fibre-optic probe through the 1.2 mm working channel of a paediatric bronchoscope (Olympus BF type 3C160, Olympus, Tokyo, Japan) (Fig. 7). The tip of the probe was gently positioned in the ASL lining in the right middle lobe bronchus under direct vision. While adequate contact of the tip of the probe against the wall of the right middle lobe was ensured, care was taken to avoid excessive mechanical pressure that may trigger ion flux across the airway lumen. The tip of the probe was held in position until accurate measurements were obtained. Investigators were blinded to pH readings during measurements, but the phase shift was visualised in real time as described in the Supplementary Information online. Measurements were performed within 10 min. Lower airway pH measurements were performed prior to bronchoalveolar lavage.

### Processing of in vivo pH measurements

Investigators were blinded to pH readings during measurements, as only the phase shift was visualised in real time. Mean pH measured for each experiment was determined visually afterwards from pH vs. time plots by two independent investigators (N.C.S. and S.M.B.) who were blinded to disease status of study subjects from whom the measurements were obtained. Examples of pH tracings over time are shown in Supplementary Fig. 3. On two occasions where mean pH values determined for a study subject differed between investigators by more than 0.1 pH units, a third investigator (A.S.) determined mean pH from the same pH vs. time plots. This measurement was then averaged with the measurement of N.C.S. or S.M.B. that was within 0.1 pH units.

### Post in vivo measurement validation of probes

After the in vivo pH measurement was completed, the probe used was returned to the pH 7.6 buffer used in the calibration at 37 °C. This was performed to indicate the stability of the calibration as well as the functionality of the probe during the measurement period. A weak correlation between the in vivo pH measurement and the post-measurement pH reading was identified by linear regression analysis as shown in Supplementary Fig. 4.

Two methods were applied to correct for probe drift before in vivo measurements. One method was to exclude all in vivo measurements from probes that did not give readings within a range of pH 7.6 ± 0.05 when placed in the pH 7.6 buffer post in vivo measurements; probes that did not return to pH 7.6 ± 0.05 post measurement were eliminated from analysis. This resulted in 20 data points being withdrawn from a total data set of 51 measurements. It is important to note that omission of these outliers did not affect study outcomes (Supplementary Fig. 5), with mean±s.d. pH in controls vs. CF 6.97 ± 0.10 and 7.01 ± 0.13 respectively (*p* = 0.33) using a two-sided *t*-test.

A second method to correct for probe drift was to calculate the gradient of pH change between calibration pre and post in vivo measurements. A corrected calibration point was then determined at the point when in vivo measurements began, and final in vivo measurements were adjusted accordingly. Correction for probe drift also did not change study outcomes (Supplementary Fig. 6), with mean±s.d. pH in controls and CF 7.00 ± 0.12 and 7.01 ± 0.13 respectively (*p* = 0.78) using a two-sided *t*-test.

### Validation of fibre-optic probes in an ex vivo setting

As the fibre-optic system relies on the detection of a change in the light signal to calculate pH, there was a theoretical possibility that the reflective properties of the airway surface in vivo might interfere with the light signal, leading to an inaccurate pH reading. To verify that the fibre-optic system was suitable to measure pH in the in vivo setting, 2–3 mL of the buffer (pH 7.6) was placed on the mucosal surface of a 10 cm × 7 cm × 0.5 cm section of bovine trachea and the pH of the buffer measured. The buffer spread out to cover the surface of the trachea in a thin layer.

Measurements were performed in triplicate at 37 °C in an incubator. The probe was placed at either a perpendicular or 45° angle to the airway surface. A micromanipulator was used to maintain the position of the probe and to ensure that the probe was immersed in the buffer solution without coming into contact with the tissue. Once again, the pH reading was accepted once it had stabilised, indicated by a variation of < 0.05 pH units in 1 min. The Mann–Whitney *U*-test was used to assess whether the positioning of the probe had any effect on the pH readout. There was no evidence to support a significant effect of probe position as shown in Supplementary Fig. 7.

### Responsiveness of probes in a biological environment

The fibre-optic probes were highly responsive to detecting a reduction in ASL pH induced by increasing ambient CO_2_ levels in the incubator from 5 to 15% (Supplementary Figs 1 and 8a). Similarly, the probes were highly responsive to detecting an increase in ASL pH induced by increasing the bicarbonate levels in the basal culture medium (Supplementary Fig. 8b). Notably, there were no differential effects seen in ASL pH between CF and non-CF in response to increasing ambient CO_2_ or to increasing culture medium bicarbonate levels (Supplementary Fig. 8c, d).

### Effect of general anaesthetics on ASL pH

In order to test if the general anaesthetic agent used during measurements, propofol, had an effect on ASL pH, propofol was added to the culture medium of human airway epithelial cell cultures at levels associated with general anaesthetics^35^. Adding propofol to basal culture medium had no effect on ASL pH (Supplementary Fig. 9).

### Bronchoalveolar lavage

BAL fluid collected at the time of bronchoscopy, but after pH measurements. Bronchoscopy and BAL were performed under general anaesthesia, using a total intravenous protocol, at a time when the children were considered to be clinically stable and not suffering from pulmonary exacerbation. Suction of pulmonary secretions was delayed until the tip of the bronchoscope was below the level of the carina to avoid upper airway contamination. Three aliquots of normal saline (1 mL/kg body weight) were instilled into the right middle lobe and retrieved using low-pressure suction. The first aliquot was sent to the laboratory for culture and the identification of bacteria and fungal elements. The remaining two aliquots were stored on ice until pooled and processed (within 3 h of collection) for assessment of inflammation.

### Measurement of inflammation

BAL fluid was analysed for inflammatory markers including total leucocyte count, differential leucocyte count and IL-8. Cell pellets were obtained from the BAL fluid samples by centrifugation, cytospins prepared from the resuspended cells and stained with Leishman’s stain for the differential cell count. IL-8 levels were determined using an enzyme-linked immunosorbent assay (BD Opt EIA, BD Biosciences, Australia) with a working range of 10–6,400 pg/mL^36–38^. Samples were measured in duplicate and were diluted to fall in the linear portion of the standard curve.

### Assessment of infection status

BAL samples from all participants (CF and non-CF) were cultured on horse blood agar (BA) (Oxoid, Thermo Fisher, Melbourne, Australia), cysteine lactose electrolyte-deficient agar (CLED) (Oxoid, Thermo Fisher), Filde’s agar (PathWest Laboratory Medicine WA Media, Perth, Australia) for isolation of *Haemophilus influenzae*, and Sabourauds’ agar with chloramphenicol (SABC) (Sigma Aldrich, Sydney, Australia) for yeast/fungi. In addition, samples were cultured on *Burkholderia cepacia* selective agar (BCSA) (Becton Dickinson, Sydney, Australia), colistin nalidixic acid agar (CNA) (Oxoid, Thermo Fisher) for *Streptococcus pneumoniae*, and mannitol salt agar (MSA) (Oxoid, Thermo Fisher) for *Staphylococcus aureus*.

Each plate was streaked with 20 μl BAL fluid. BA, CLED, BCSA, CNA and SABC agars were incubated aerobically for 48 h at 35 °C in 5% CO_2_. Cultures were read at 24 and 48 h. SABC was further incubated for 12 days in air at 28 °C to examine fungal growth. MSA agar was incubated aerobically for 48 h at 35 °C in air. Filde’s agar was incubated anaerobically for 48 h at 35 °C to inhibit the growth of *Pseudomonas* and thus aid recovery of *H. influenzae*.

Colonies of suspected pathogens growing from culture media were identified using various conventional tests including Gram stain, catalase, oxidase, Phadebact (MKL Diagnostics, Sollentuna, Stockholm), Staphylococcal latex test, API™ (BioMérieux, Marcy-l'Étoile, France) and automated identification by Vitek (BioMérieux).

### Epithelial cell culture for fibre-optic probe experiments

Epithelial cell cultures were derived from lower airway brushings from children with and without CF. Children with CF were recruited during their clinically directed annual surveillance visit while children without CF were recruited after hospital admission for elective non-respiratory-related surgery. Demographics of the patients recruited for epithelial cell culture studies are provided in Supplementary Table 1. Informed consent was obtained from each participants’ legal guardian at the time of recruitment. Ethical approval for the collection of airway epithelial cells was granted by the Princess Margaret Hospital Human Research Ethics Committee with regards to patients with CF, and the St John of God Health Care Human Research Ethics Committee for participants without CF. Epithelial cells obtained from the cytological brushing were used to establish a conditionally reprogrammed primary cell culture. Conditionally reprogrammed cells were used to seed 6.5 mm Transwell Clear supports (Corning) in a well-defined airway culture media^39^. After confluence was reached (3–5 days), cultures were maintained at air–liquid interface (ALI). Experiments were performed on ALI cultures 28 days after the media at the apical surface were removed.

### In vitro pH measurements with fibre-optic probes

At 24 h prior to the experiment, accumulated mucus and cellular debris on the apical surface of the ALI cultures were removed by three gentle washes with PBS for 10 min each at 37 °C. For one ALI culture per patient, standard media were replaced by high HCO_3_^−^ media. On the following day, cultures were transferred to a tissue culture incubator housing the pH measuring equipment, where typical cell culture conditions were maintained (5% CO_2_, 37 °C, 95% humidity). The fibre-optic probes were calibrated according to the same procedure described for the in vivo pH measurements. A micromanipulator (World Precision Instruments, USA) was used to remotely lower the fibre-optic probe into the ASL at 5 µm increments. A drop in light signal intensity was noted at the point of contact of the probe tip with the ASL whereupon probe position was fixed. Experiments were performed by taking four replicate measurements for each experimental condition as shown in Supplementary Table 2. For ALI cultures receiving propofol treatment, 10 µL of a 1000 µg/mL working solution of Propofol-Lipuro 1% (B Braun, Australia) was added to the basolateral media at least 15 min prior to pH measurement. For the duration of each measurement, real-time pH values were logged at 5 s intervals using pH logger software as per in vivo pH measurements.

### The in vitro fibre-optic probe pH data analysis

Logged data obtained prior to equilibration of carbon dioxide concentration within the incubator were excluded from analysis. Measurements were taken after the probes made contact with the ASL and as soon the signal stabilised, i.e., when there was <0.01 pH unit change over a 1 min period. Replicate measurements were excluded if disturbances to the apical layer of the cell culture surface were detected by light microscopy. Median values of the accepted pH replicates were used as a representative value for each ALI culture.

### Cell culture for conventional micro-pH probe measurements

Human tracheobronchial epithelial cells from normal donors and CF patients were obtained from the University of North Carolina (UNC)-CF Tissue Procurement Core under the auspices of protocols approved by the UNC institutional review board on the protection of the rights of human subjects. Normal epithelial cells were derived from donor lungs and excess tissue of the recipient lung at the time of transplantation, and CF tissues from autopsy- and lung transplant-derived tissues. Cells were harvested by enzymatic digestion using well-documented techniques^40^: donated lung tissue samples were successively dissected to remove surrounding connective tissue and obtain 1 × 2 cm longitudinal tissue sections. A series of antibacterial washing steps were applied to clean the tissue segments and minimise contamination risk. Lung tissue segments were then treated with a Protease/DNase solution for 48 h at 4 °C followed by treatment with 10% foetal bovine serum to terminate the enzymatic digestion. Disaggregated human airway epithelial cells were seeded on 12 mm diameter Transwell Clear supports (Corning) at a density of 2.5 × 10^5^/cm^2^ in a well-defined airway cell media^40^. After confluence was reached (3–5 days), cultures were maintained at ALI until fully differentiated (~4 weeks after seeding).

### The in vitro pH measurements with conventional micro-pH probe

At 24 h before the study, accumulated mucus and cellular debris on the CF and non-CF cultures were removed from the luminal surface by three gentle washes with PBS for 10 min each. On the following day, cultures were placed in a tissue culture incubator outfitted with our custom pH measuring system. Throughout the assay, appropriate environmental conditions were maintained (i.e., 5% CO_2_, 37 °C, 95% humidity). pH was measured using a micro-pH meter (0.65 mm diameter; Innovative Instruments, Inc., USA) which does not require full immersion for measuring pH. During the assay, a micromanipulator (Siskiyou, USA) was used to remotely advance the micro-pH probe into the thin airway surface layer. Positioning of the probe was visually monitored and confirmed using a magnified camera (90X; Dino-Lite) outfitted with a right-angle objective. ASL pH values were obtained once steady-state values were obtained, typically within 1–2 min following immersion of the probe into the ASL. The pH meter was regularly calibrated with pH standards (Fisher Scientific).

### ATP12A overexpression experiments

Calu-3 cell lines: Bronchial adenocarcinoma Calu-3 cells are a model of polarised human airway epithelia^27^. Calu-3 cells (ATCC) were transfected with puromycin-encoding pQCXIP (Clontech) retroviral vectors, empty (control) or encoding the complementary DNA of human ATP12A^9^, following established techniques in our lab^41^. Cells were selected with puromycin (1 µg/mL), and differentiated on permeable supports to ALI culture conditions for 1 week^27^. Calu-3 cells were subjected to luminal pH measurements using a potentiometric microsensor pH electrode in thin-film, unperturbed ASL, as described above. Luminal pH was recorded for ~60 min, every 2 s, after equilibration, in three independent cultures per experimental condition.

Primary human airway epithelial cells: pQCXIP empty vector or encoding human ATP12A were transfected into non-differentiated passage 2 primary human airway epithelial cells cultured on plastic support according to protocols established in our lab. Human cells were puromycin selected and plated onto collagen-coated permeable supports for ALI differentiation^42^. Cells were cultured for 4 weeks and evaluated for luminal pH using a surface contact pH microelectrode in thin-film, unperturbed ASL, as described for Calu-3 cells. Human epithelial cells were obtained from excess transplant tissue from normal donors following the human subject regulations of the University of North Carolina at Chapel Hill.

### Statistical analysis

Student’s *t*-test was used to compare groups where data were normally distributed. The Mann–Whitney *U*-test was used to compare groups where data were not normally distributed. Spearman’s rank correlation was used to study associations between lower airway pH and inflammation.

### Data availability

All the data are available from the corresponding author on reasonable request.

## Electronic supplementary material

Supplementary Information
