# Supplementary material for: Airway surface liquid pH is not acidic in children with cystic fibrosis
Source: Nat Commun. 2017 Nov 10;8:1409. doi: 10.1038/s41467-017-00532-5 (PMC5680186; doi:10.1038/s41467-017-00532-5)
Supplement: Supplementary file 1 — Supplementary Information [file 41467_2017_532_MOESM1_ESM.pdf]

File name: Supplementary Information
Description: Supplementary figures and supplementary tables.

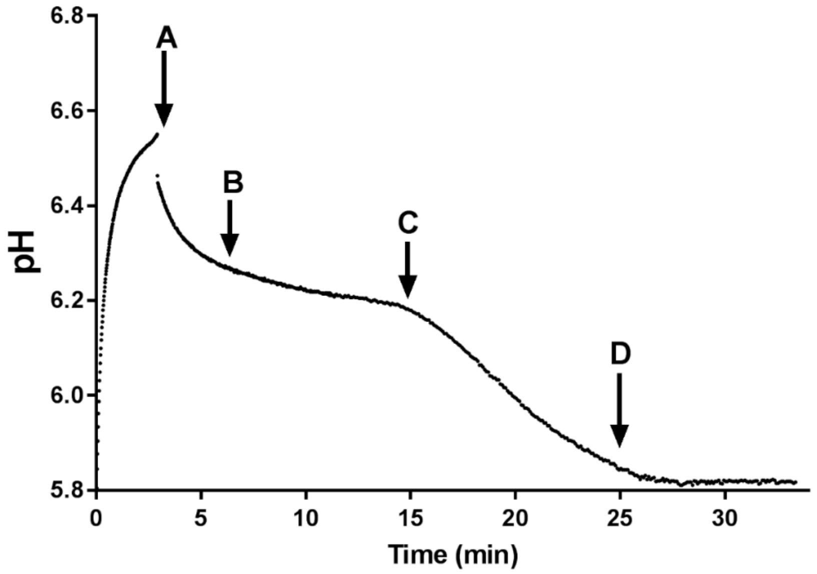


**Supplementary Figure 1**. *In vitro* ASL pH measurement trace vs. change in ambient $CO_2$ over time. Sensor hits ASL of cell culture at point A. $CO_2$ level reaches equilibrium at 5% at point B. $CO_2$ level set point of incubator changed to 15% at point C, leading to $CO_2$ injection. $CO_2$ level reaches equilibrium at 15% at point D.

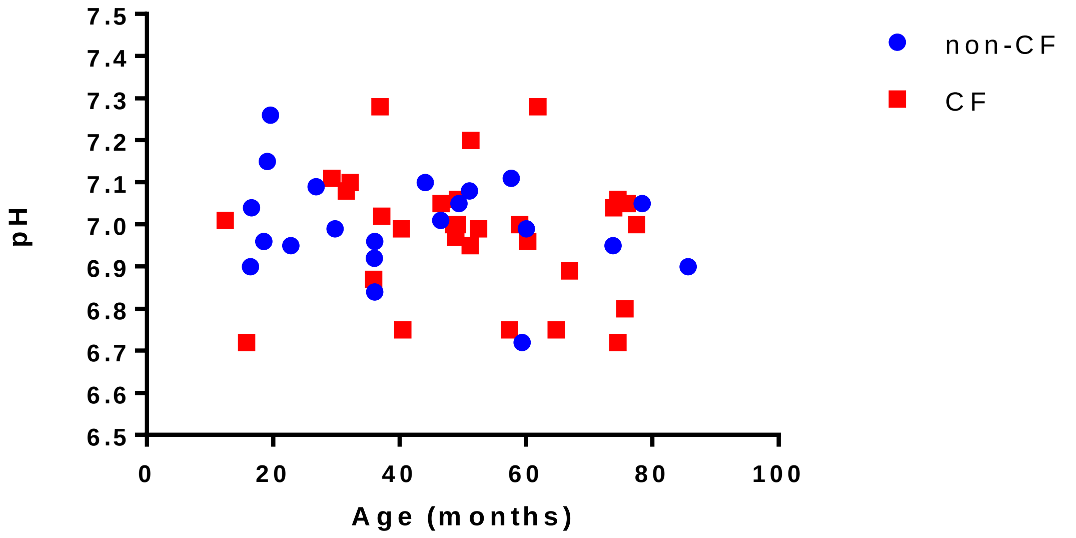


**Supplementary Figure 2.** *In vivo* measurements of ASL pH vs. age of the patient at the time of measurement. Linear regression analysis demonstrated no significant relationship between ASL pH and the age of the patient for either children with ($r^2$=0.01; p=0.58; n=30) or without CF ($r^2$=0.05; p=0.31; n=21).

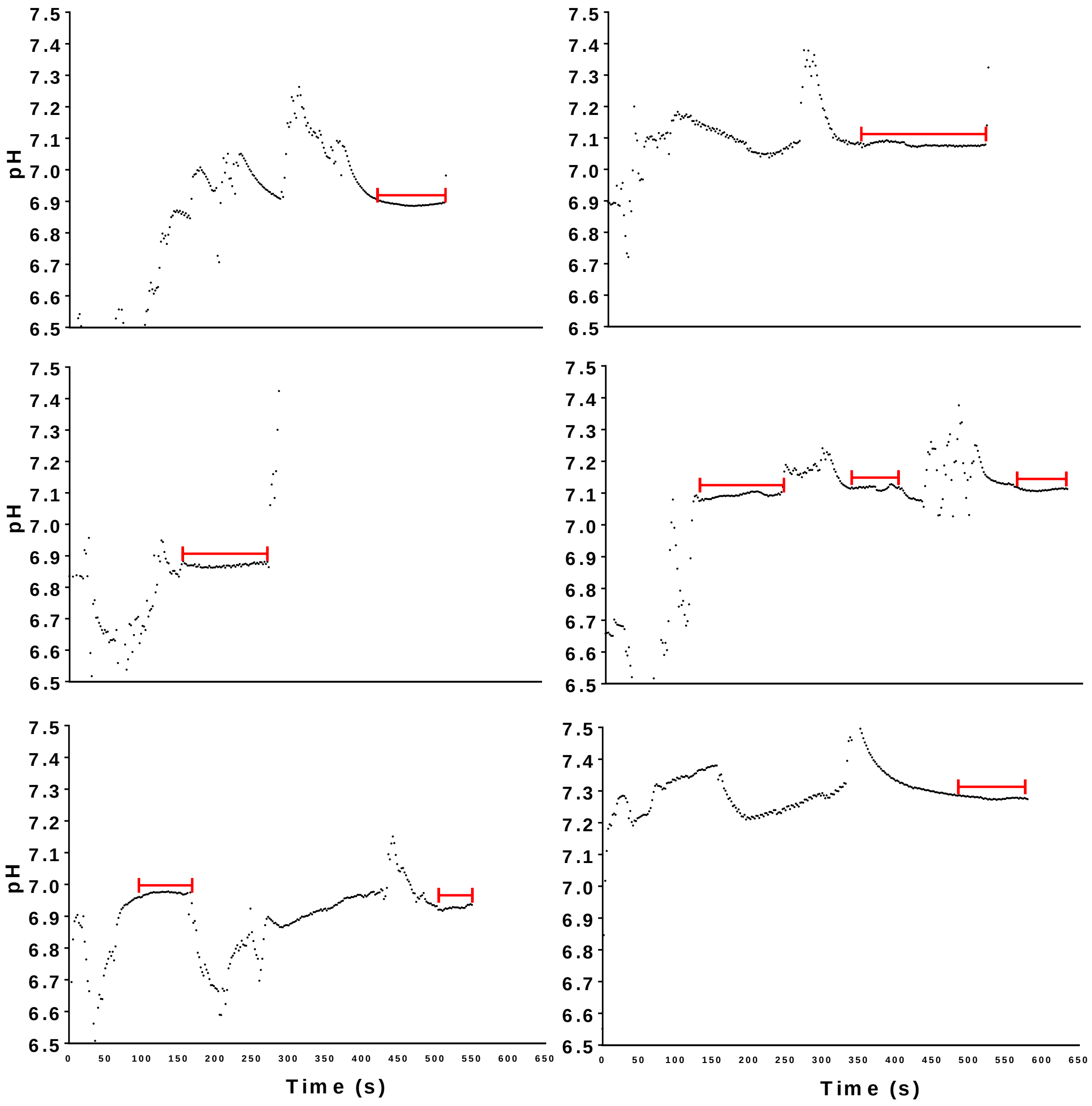


**Supplementary Figure 3.** Examples of *in vivo* transbronchoscopic pH measurements in 6 different patients. Solid lines correspond to probe contact with the airway surface. Red markings depict plateaus that an individual investigator identified and averaged to determine airway surface liquid (ASL) pH.

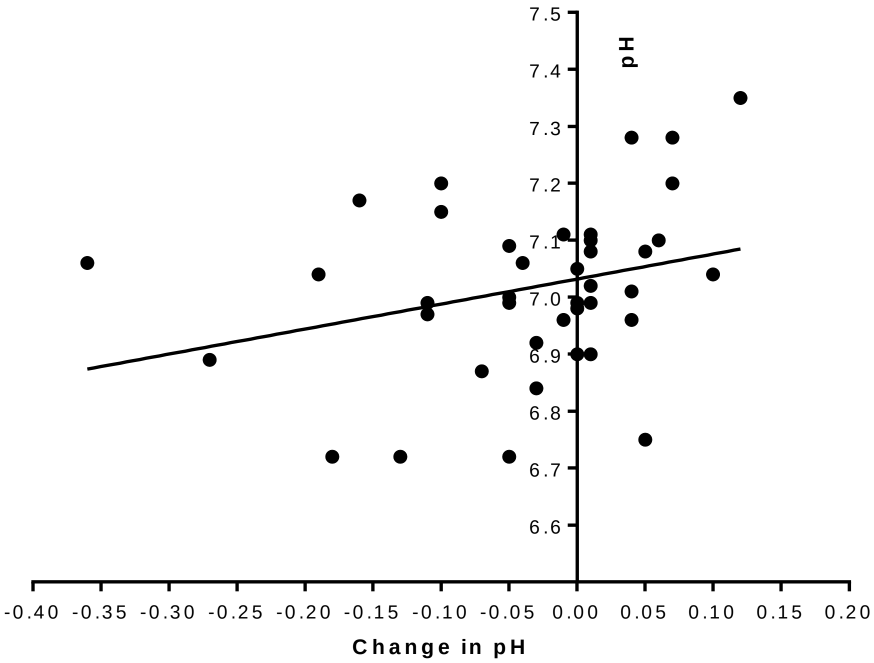


**Supplementary Figure 4.** *In vivo* pH measurement versus change in pH from 7.6 post-measurement. After *in vivo* pH readings were obtained, fibre-optic probes were returned to the pH 7.6 buffer used in the calibration and the difference from pH 7.6 calculated. Linear regression analysis demonstrated a relationship between the change in pH at the validation step and the *in vivo* measurement recorded ($r^2$ =0.18, p= 0.001, n=56).

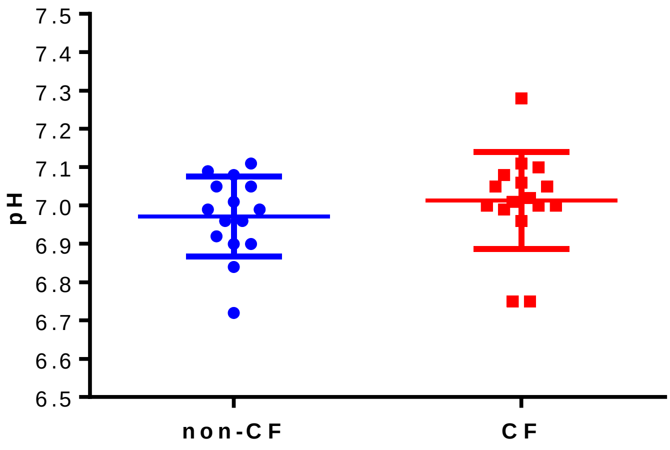


**Supplementary Figure 5.** Comparison of lower airway surface liquid pH measurements in children with and without cystic fibrosis after exclusion of measurements from pH probes that failed the post-measurement validation step. No difference was found in lower airway pH between non-CF (n=15) and CF (n=16) groups with mean±SD pH in controls vs. CF, 6.97±0.10 and 7.01±0.13 respectively, p =0.33. Two-sided t-test was used to compare CF and non-CF groups. Data presented as individual measurements and mean±SD.

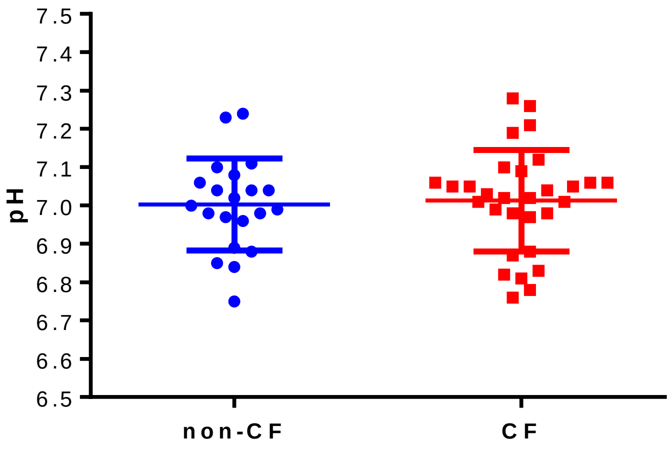


**Supplementary Figure 6.** Comparison of lower airway surface liquid pH measurements in children with and without cystic fibrosis after correcting for probe drift using corrected calibration point. No significant difference was found between the groups with mean±SD pH in controls (n=21) and CF (n=30), 7.00±0.12 and 7.01±0.13 respectively, p=0.78. Two-sided t-test was used to compare CF and non-CF groups. Data presented as individual measurements and mean±SD.

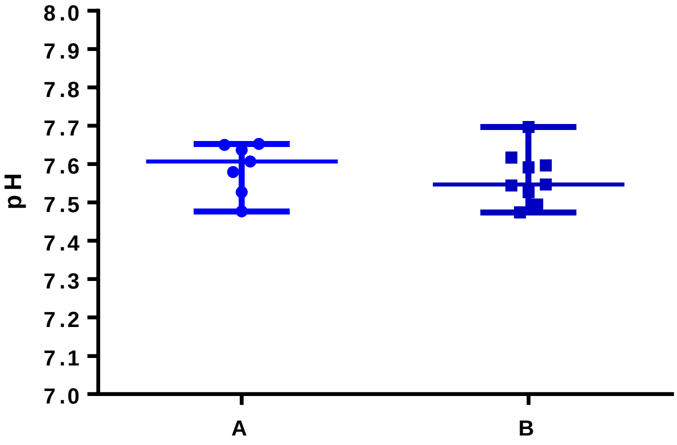


**Supplementary Figure 7.** Measurement of a pH 7.6 buffer using fibre-optic probes placed at (A) a 45$^{o}$ angle (n = 7) and (B) a perpendicular angle relative to the mucosal tracheal surface (n = 9). Median (range) of pH measurements were 7.61 (7.48-7.65) and 7.55 (7.47-7.70) in groups A and B respectively. All measurements were performed in triplicate. A two-sided Mann-Whitney test demonstrated that the positioning of the probe had no significant effect on the pH reading (p=0.46). Data presented as median (range).

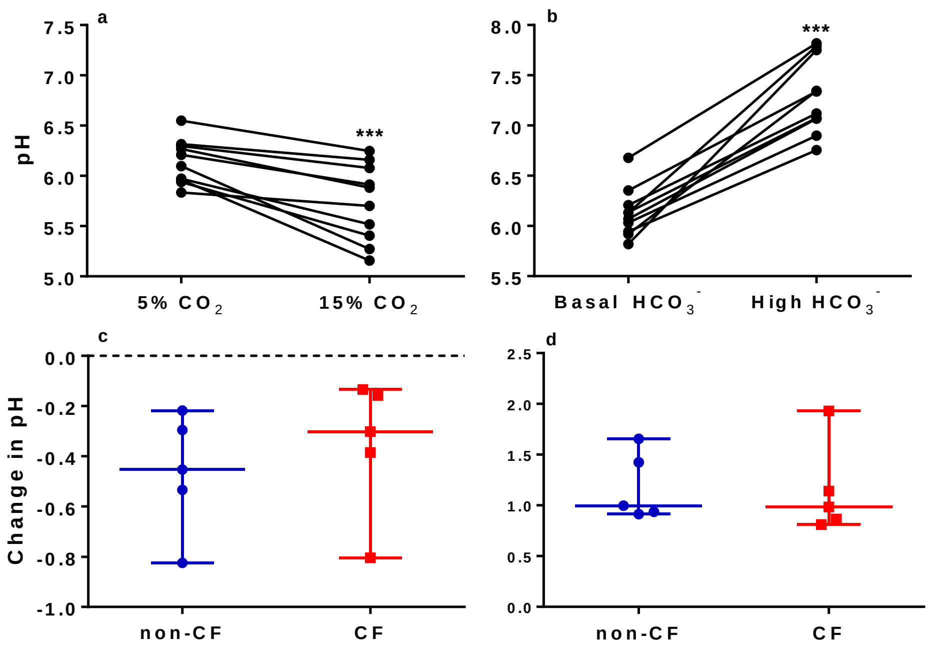


**Supplementary Figure 8.** Airway surface liquid pH in airway epithelial cells cultures. **(a)** Paired t-test demonstrated a significant decrease in ASL pH when epithelial cell cultures (n=10; 5 CF and 5 non-CF) were exposed to 15% $CO_2$ ($p<0.001$). **(b)** Paired t-test comparing epithelial cell culture (n=10; 5 CF and 5 non-CF) at basal $HCO_3^-$ and high $HCO_3^-$ media concentrations showed a significant increase ($p<0.001$) in ASL pH. There were no significant differences in the effect of high $CO_2$ **(c)** or $HCO_3^-$ **(d)** levels on ASL pH between CF (n=5) and non-CF (n=5) epithelial cell cultures. Figures (a) and (c) describe the same experiment while figures (b) and (d) reflect the same experiment. All data presented as median (range), ***$p<0.001$. CF cell cultures obtained from children with clinical CF disease -genotypes: three of the cultures were of the p.Phe508del/p.Phe508del genotype with cultures generated from different patients. Two of the cultures were of the p.Gly85Glu/p.Arg 352Gln genotype with cultures generated from the same patient at different sampling time points. These experiments also demonstrate that baseline ASL pH in a cell culture model is highly dependent on experimental factors such as the $HCO_3^-$ concentration in the culture medium.

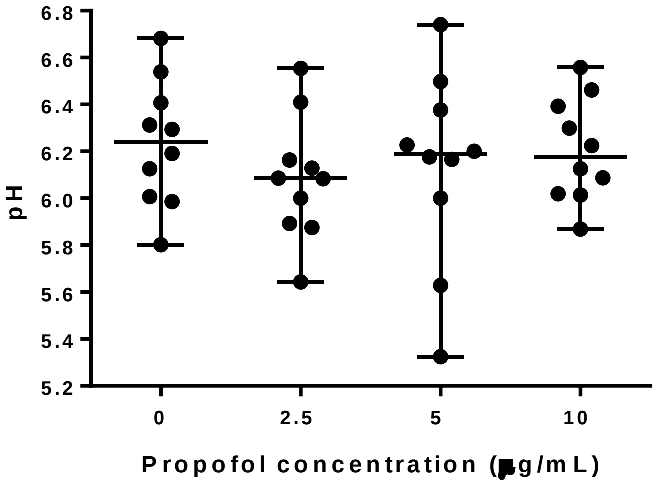


**Supplementary Figure 9.** Airway surface liquid pH after the addition of propofol to the basal media of airway epithelial cell cultures. No differences between 0 µg/mL propofol (n=10) and 2.5 µg/mL (n=10; p=0.23), 5 µg/mL (n=10; p=0.62) or 10 µg/mL (n=10; p=0.77) were determined by Wilcoxon tests. Data presented as median (range).

| Sample | Cohort | Age (months) | Sex | CFTR mutation |
|---|---|---|---|---|
| 1 | AREST CF | 34.9 | M | p.Phe508del/ p.Phe508del |
| 2 | AREST CF | 3.4 | M | p.Phe508del/ p.Phe508del |
| 3 | AREST CF | 23.4 | F | p.Phe508del/ p.Phe508del |
| 4# | AREST CF | 5.9 | F | p.Gly85Glu/p.Arg 352Gln |
| 5# | AREST CF | 11.4 | F | p.Gly85Glu/p.Arg 352Gln |
| 6 | WAERP | 36.4 | F | n/a |
| 7 | WAERP | 30.1 | M | n/a |
| 8 | WAERP | 33.0 | F | n/a |
| 9 | WAERP | 35.5 | M | n/a |
| 10 | WAERP | 19.9 | F | n/a |

**Supplementary Table 1.** Patient demographics for primary airway epithelial cell cultures used in fibre-optic probe ASL pH experiments. #Cultures generated from the same patient at different sampling times.

| Experimental condition | Basolateral media $HCO_3^-$ concentration (mM) | Atmospheric $CO_2$ concentration (%) | Basolateral media Propofol concentration (µg/mL) |
|---|---|---|---|
| Basal | 27.95 | 5 | 0 |
| High $HCO_3^-$ | 83.85 | 5 | 0 |
| High $CO_2$ | 27.95 | 15 | 0 |
| Propofol treatment | 27.95 | 5 | 10 |

**Supplementary Table 2.** Experimental conditions for *in vitro* pH experiments in human bronchial epithelium air-liquid interface cultures.
